# Boosting with heterologous vaccines effectively improves protective immune responses of the inactivated SARS-CoV-2 vaccine

**DOI:** 10.1080/22221751.2021.1957401

**Published:** 2021-08-18

**Authors:** Jialu Zhang, Qian He, Chaoqiang An, Qunying Mao, Fan Gao, Lianlian Bian, Xing Wu, Qian Wang, Pei Liu, Lifang Song, Yaqian Huo, Siyuan Liu, Xujia Yan, Jinghuan Yang, Bopei Cui, Changgui Li, Junzhi Wang, Zhenglun Liang, Miao Xu

**Affiliations:** National Institutes for Food and Drug Control, Beijing, People’s Republic of China

**Keywords:** Heterologous, prime-boost, inactivated, SARS-CoV-2, vaccine, neutralizing antibody, T cell response, IgG subtypes

## Abstract

Since the outbreak of COVID-19, a variety of vaccine platforms have been developed. Amongst these, inactivated vaccines have been authorized for emergency use or conditional marketing in many countries. To further enhance the protective immune responses in populations that have completed vaccination regimen, we investigated the immunogenic characteristics of different vaccine platforms and tried homologous or heterologous boost strategy post two doses of inactivated vaccines in a mouse model. Our results showed that the humoral and cellular immune responses induced by different vaccines when administered individually differ significantly. In particular, inactivated vaccines showed relatively lower level of neutralizing antibody and T cell responses, but a higher IgG2a/IgG1 ratio compared with other vaccines. Boosting with either recombinant subunit, adenovirus vectored or mRNA vaccine after two-doses of inactivated vaccine further improved both neutralizing antibody and Spike-specific Th1-type T cell responses compared to boosting with a third dose of inactivated vaccine. Our results provide new ideas for prophylactic inoculation strategy of SARS-CoV-2 vaccines.

## Introduction

The phase 3 clinical data of various COVID-19 vaccines based on different platforms have been recently published one after another. The advanced mRNA vaccines BNT162b2 and mRNA-1273 both reported >90% efficacy [1,2]. Adenovirus vectored vaccines developed by AstraZeneca [3], Gamaleya Research Institute [4] and Cansino Biologics showed an efficacy of 70.4%, 91.6% and 65.7%, respectively. The recombinant subunit vaccine NVX-CoV2373 mediated an overall 89.3% protection against COVID-19 symptoms [5]. While inactivated vaccines developed by Sinopharm (Beijing) and Sinovac showed 79.34% and 50.38% efficacy, respectively [6]. Although all these vaccine candidates met the 50% efficacy criterion laid out for emergency use by multiple National Regulation Authorities and WHO [7–9], and have been approved in several countries, the immunogenicity and protective efficacy of these vaccines developed in urgency still needs to be improved further.

An ideal COVID-19 vaccine should possess the ability to induce both protective antibodies and T cell immune responses for clearing the virus. For example, neutralizing antibodies (NAbs) targeting the Spike protein elicited by the vaccine can potentially block the cellular entry process of the virus, while activation of T cells, especially cytotoxic T cells, can play an important role in eliminating virus infected cells [10]. Data from clinical research have shown that the immune responses of vaccines vary significantly. The geometric mean titres (GMTs) of NAbs induced by three inactivated vaccines developed in China were 64 for CoronaVac [11], 282.7 for BBIBP-CorV [12], and 247 for COVID19 vaccine developed by Wuhan institutes of Sinopharm [13]. One reason for this discrepancy could be attributed to the lack of uniformity in materials and methods used for immunogenicity measurement. Another reason could be the differences in manufacturing processes and equipment used for developing the inactivated vaccines, leading to differences in antigenicity of the vaccines. The differences are more pronounced between vaccines from different platforms. Recently, we analysed the neutralizing antibody data for vaccinated and convalescent sera, deducing the ratios of NAb GMTs of vaccinated sera to convalescent sera [14]. The results of the analysis revealed that vaccinated/convalescent ratios of inactivated vaccines fall in the range of 0.27–1.11, which are relatively lower than those of other vaccine candidates which fall between 1.41 and 4.13. Furthermore, nucleic acid vaccines [15–18] and adenovirus vectored vaccines [19–21] confer additional advantage *via* inducing more robust cellular immune responses because vaccination with these types of vaccines involves the synthesis and presentation of viral peptides on the surface of cells along with MHC molecules. However, different vaccines of these types also show significant variations in eliciting cellular immune responses, suggesting that there is room for further improvement.

Heterologous prime-boost strategy [22,23], especially a combination of exogenous vaccines (Targeted antigens were delivered into cells like protein-based vaccines, inactivated vaccines, and so on) and endogenous vaccines (Targeted antigens were expressed in cells like nucleic acid-based vaccines-mRNA vaccine, DNA vaccine, virus vectored vaccine, and so on), has been shown to effectively improve the immunogenicity of HIV-1, influenza, SARS-CoV-2 vaccines [24–27]. Several regulatory agencies have already begun contemplating putting into practice the concept of heterologous prime-boost strategy or mixed vaccination regimen. Data on the compatibility of different vaccines and evaluation of the beneficial effects of a heterologous prime-boost strategy in clinical trials is necessary to support this endeavour.

Inactivated vaccines, such as BBIBP-CorV (Sinopharm Beijing), inactivated vaccineWIBP (Sinopharm Wuhan), Coronavac (Sinovac) and BBV152 (Bharat Biotech), have been approved for emergency use in several nations. Subsequently, a large portion of the population of the world has completed a two-dose emergency vaccination. It should be noted that the antibody level would gradually decrease over time [28], and the long-term protective effect of the inactivated vaccine still needs long-term monitoring. To sustain and prolong the duration of protection, a third dose of inactivated vaccine or a heterologous vaccine could be necessary. This study explores the utility of homologous and heterologous prime-boost strategies to improve the protective immune responses against SARS-CoV-2 in a mouse model. The implications for the findings in vaccine application are discussed.

## Materials and methods

### Animals and vaccines

Six- to eight-week-old female-specific pathogen-free BALB/c mice used in the study were provided and maintained by Chinese National Institutes of Food and Drug Control. Four SARS-CoV-2 vaccine candidates developed by different platforms were used in this study, including inactivated vaccine, adenovirus vectored vaccine, recombinant protein vaccine and mRNA vaccine. The vaccines used in the study were donated by different developers and manufacturers, and were vaccinated by 1/5 corresponding human dose.

### ELISA for estimating spike-specific IgG

ELISA was conducted to determine the titres of serum binding antibodies to SARSCoV-2 spike as described in the previous study [26]. Ninety-six-well EIA/RIA plates were coated with SARS-CoV-2 spike protein at 1 μg/ml at 4°C overnight. Plates were washed 3 times with PBST (PBS containing 0.05% Tween-20) to remove unbound spike protein and then blocked with 10% Fetal Bovine Serum in 0.5% PBST for 2 h at 37°C. 10-fold serially diluted test samples were added to the wells and incubated for 1 h at 37°C. Then, the plates were washed and incubated with 1:5000 diluted goat anti-mouse IgG secondary Abs (HRP labelled) (ZSGB-BIO, cat#ZB2305) followed by detection with substrate (Wantai BioPharm, cat#N20200722) at 450 and 630 nm. For IgG subtypes detection, goat antimouse IgG1 secondary Abs (HRP labelled) (Abcam, cat#ab97240) and goat anti-mouse IgG2a secondary Abs (HRP labelled) (Abcam, cat#ab97245) were used as secondary Abs and diluted by 1:10,000. The endpoint of serum antibody titres was determined as the reciprocal of the highest dilution that was 2.1-fold higher than the optical absorbance value of the negative control.

### Serum neutralization assay

The serum neutralizing antibodies to SARS-CoV-2 were measured using live and pseudo SARS-CoV-2 virus and the results were expressed as 50% inhibitory dilution (EC50) of serum. The neutralizing antibodies to live SARS-CoV-2 (virus strain SARSCoV-2/human/CHN/CN1/2020, GenBank: MT407649.1) were quantified using a micro cytopathogenic effect assay with a minimum eight-fold dilution [29]. The neutralization capacities were also measured by pseudo-virus (GenBank: MN908947, optimized for human cell expression) as described in previous work [30].

### IFN-γ ELISPOT assay

IFN-γ enzyme-linked immunosorbent spot (ELISpot) assay was performed using the mouse IFN-γ ELISPOT kit (BD, cat#551083). First, freshly isolated splenocytes were collected and co-cultured with four separate peptide pools spanning SARS-CoV-2 spike protein for 20 h at 2 × 10^5^ cells per well. The concentration for each peptide was 5 μg/ml. Peptide pools were generated as follows: a panel of consecutive 15-mer peptides overlapped with nine amino acids were synthesized spanning the whole spike protein and grouped into four pools: S1-non RBD (aa:1-324, 577-654; 67 peptides), S1-RBD (aa:325,576; 42 peptides), S2-1(aa: 655-960; 51 peptides), S2-2(aa:961-1273; 51 peptides). After stimulation, the supernatants were collected and incubated with plates coated with IFN-γ detecting antibodies. Spots representing IFN-γ producing cells were enumerated using an ELISPOT reader (ChampSpot III Elispot Reader, Saizhi, Beijing, China). The final value was calculated by subtracting the background value from the measured values.

### MSD Th1/Th2 cytokine profiling

Supernatants of 2 × 10^5^ splenic lymphocytes were collected after stimulation with 5 μg/ml peptide pools spanning SARS-CoV-2 spike protein. Supernatants stimulated by four peptide pools were pooled together according to different samples. Then supernatants were diluted 1:2 and measured by a V-PLEX Proinflammatory Panel 1 (mouse) Kit (MSD, cat# K15048D-1). The concentration of each sample was calculated using a standard curve. The concentration of unstimulated samples was subtracted from the levels of stimulated samples.

### Statistical analysis

The antibody titres were transformed into log10 titres for the calculation of GMTs. All statistical analyses were conducted using GraphPad Prism 7.0 (GraphPad Software, Inc). To compare the mean of different groups, One-way ANOVA was performed for multiple groups (>2) comparison; two-tailed student’s t-test was performed for two groups comparison.

## Results

### Humoral immune responses elicited by one dose of inactivated, recombinant subunit, adenovirus vectored or mRNA vaccine in mice

**Figure 1.**
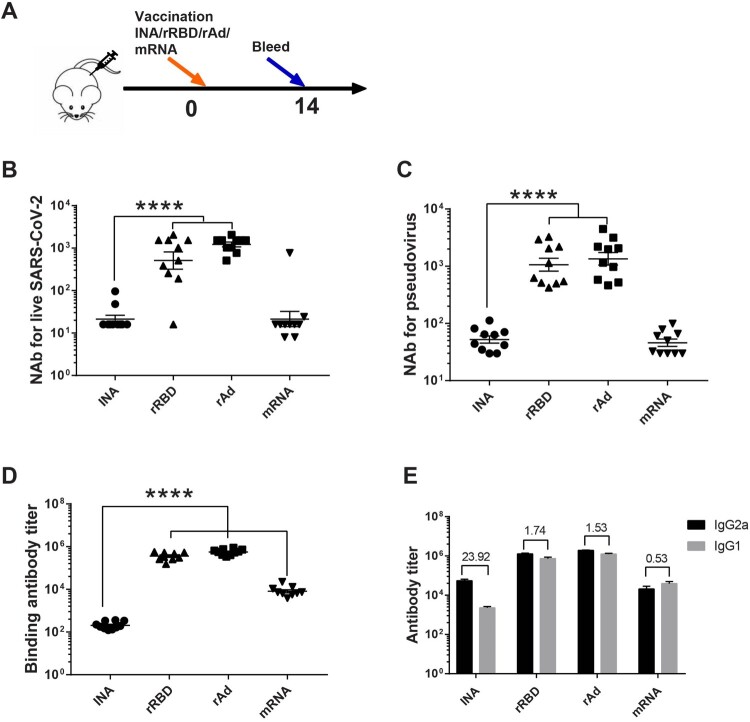
Humoral immune responses induced by different vaccine platforms. (A) Schematic representation of experimental protocol and immunization groups. Mice in four groups were immunized with single dose of different COVID-19 vaccines: INA, rAd, rRBD and mRNA. (INA: inactivated vaccine, rAd: recombinant Ad5 vectored vaccine, rRBD: recombinant RBD vaccine, mRNA: mRNA-based vaccine). The dosages used for this experiment were 1/5 human dose for INA (0.8 μg), rRBD (10 μg), rAd (1 × 10^10^ vp) and mRNA (5 μg). (B, C). Sera NAb titres measured by live SARS-CoV-2 virus (B) and pseudovirus (C), the neutralizing antibody (NAb) concentrations were expressed as 50% inhibitory dilution (EC50) of serum. (D). Spike-specific binding IgG titres were measured by ELISA. (E). The titres of IgG subtypes were measured by ELISA, the ratios of IgG2a/IgG1 for each group were calculated and shown in chart. *N* = 10 per group, one spot represents one sample. One-way ANOVA was performed for B, C and D; two-tailed student’s *t*-test was performed for E. Bars represent the mean ± SEM, ****p < 0.0001.

COVID-19 vaccines have been shown to evoke a wide range of immune responses. The responses induced by individual vaccines are hard to compare because the studies have been done under different conditions. To enable direct comparison, we administered four different kinds of vaccines: inactivated vaccines (INA) (manufacturer 1), recombinant RBD vaccine (rRBD) (manufacturer 2), Ad5-vectored adenovirus vaccines (rAd) (manufacturer 3) and mRNA vaccine (mRNA) (manufacturer 4) in mice and studied the characteristics of the immune responses elicited by them under identical conditions. rRBD used in this study was a dimeric RBD protein vaccine, rAd was a type 5 adenovirus- vectored vaccine expressing full-length S protein, while mRNA vaccine expressing RBD was encapsulated in lipid nanoparticles. BALB/c mice were immunized with one dose of each vaccine via intramuscular injection. Two weeks after the immunization, the level of binding antibody and neutralizing antibody (NAb) against live virus as well as pseudovirus were measured. Our results showed that NAb GMTs against live SARS-CoV-2 were significantly higher in rAd (1218.57) and rRBD (509.34) group than INA (21.36) and mRNA (21.36) group. A similar trend was found in NAb titres tested using pseudovirus. The NAb GMT against pseudovirus elicited by one dose of rRBD, rAd, INA and mRNA vaccine was 1061.95, 1348.18, 52.29 and 45.86, respectively. NAb GMTs elicited by INA group against live virus and pseudovirus were comparable with mRNA group (*p* > 0.05). To estimate the binding antibody levels induced by different kinds of COVID-19 vaccines, we measured the total Spike-specific IgG and the IgG subtypes in these four groups. The total spike-specific IgG GMT induced by INA was 204.57, lower than those measured in rRBD (*p* < 0.0001), rAd (*p* < 0.0001) and mRNA (*p* < 0.0001). For IgG subtypes, we measured IgG1 and IgG2a (Figure 1(E)). The IgG2a were significantly higher in rAd and rRBD group, with a GMT of 1,912,136 and 1,272,338, followed by INA (54,387) and mRNA (20,597). The IgG2a/IgG1 ratio may reflect the skewing of T helper type-2 (Th2) and T helper type-1 (Th1) responses. In our results, the IgG2a/IgG1 ratio in INA group was 23.92, higher than rRBD (1.74), rAd (1.53) and mRNA (0.53), indicating the Th1/Th2 type responses were more balanced in INA group, though the humoral response induced by INA was relatively lower.

### Spike-specific T cell responses elicited by one dose of inactivated, recombinant subunit, adenovirus vectored or mRNA vaccine in mice

**Figure 2.**
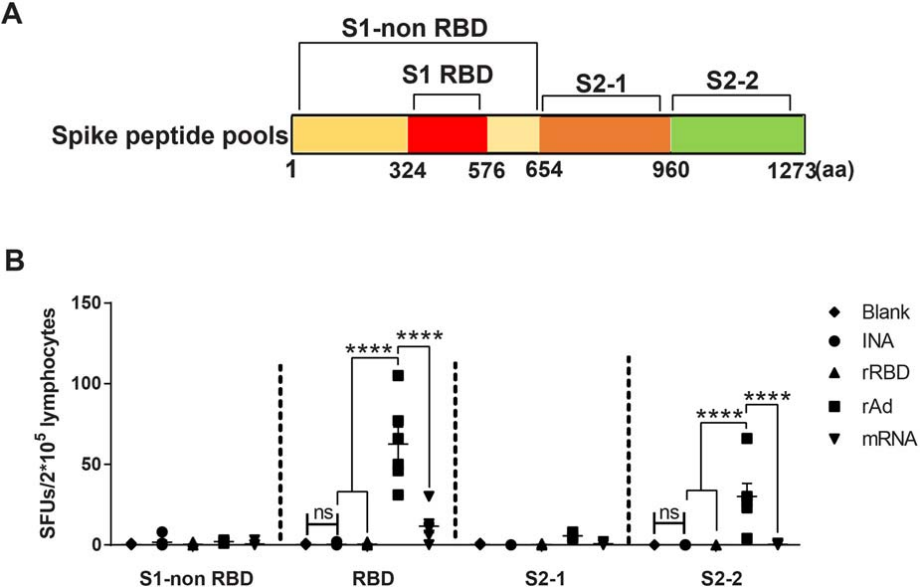
SARS-CoV-2 Spike-specific T cell responses induced by different vaccine platforms measured by INF-γ ELISPOT assay (A). Peptides spanning full length spike were synthesized and divided into four peptide pools: S1-non RBD (aa: 1-324, 577-654), S1-RBD (aa: 325-576), S2-1(aa: 655-960), S2-2(aa: 961-1273). (B). Mice were sacrificed for measuring T cell responses. Isolated lymphocytes were stimulated with 4 spike peptide pools, and the IFN-γ secreting cells were quantified by ELISPOT assay. *N* = 6 per group, one spot represents one sample. One-way ANOVA was performed for comparison. Bars represent the mean ± SEM, ns: *p* > 0.05, **p < 0.01, ***p < 0.001, ****p < 0.0001.

To characterize the T cell responses induced by COVID-19 vaccines developed by different platforms, splenic lymphocytes were collected from groups aforementioned in Figure 1 and the number of IFN-γ secreting T cells were measured post stimulation with four peptide pools spanning the Spike protein (Figure 2(A)). The results showed that S1-RBD (aa: 325-576) and S2-2 (aa: 961-1273) were the most recognized peptide pools (Figure 2(B)). For INA and rRBD group, nearly no IFN-γ secreting T cells were detected against all four peptide pools. On the contrary, rAd and mRNA vaccine both successfully elicited spike-specific T cell responses. rAd vaccinated splenocytes are stimulated by four peptide pools with SFUs/2 × 10^5^ cells of 2 for S1-non RBD, 62.5 for RBD, 5.67 for S2-1, and 30 for S2-2, respectively. mRNA vaccinated splenocytes only could be stimulated by RBD peptide pool with mean SFUs/2 × 10^5^ cells of 11.6. The T cells responses against S1-RBD in rAd group were significantly higher than other groups (*p* < 0.0001). Thus, rRBD, rAd and mRNA all could elicit specific T cell responses. Amongst, rAd vaccine showing the highest activation of T-cells.

### Heterologous prime-boost with two doses of inactivated vaccine followed by either recombinant RBD, adenovirus-vectored or mRNA vaccine improves humoral immune responses

**Figure 3.**
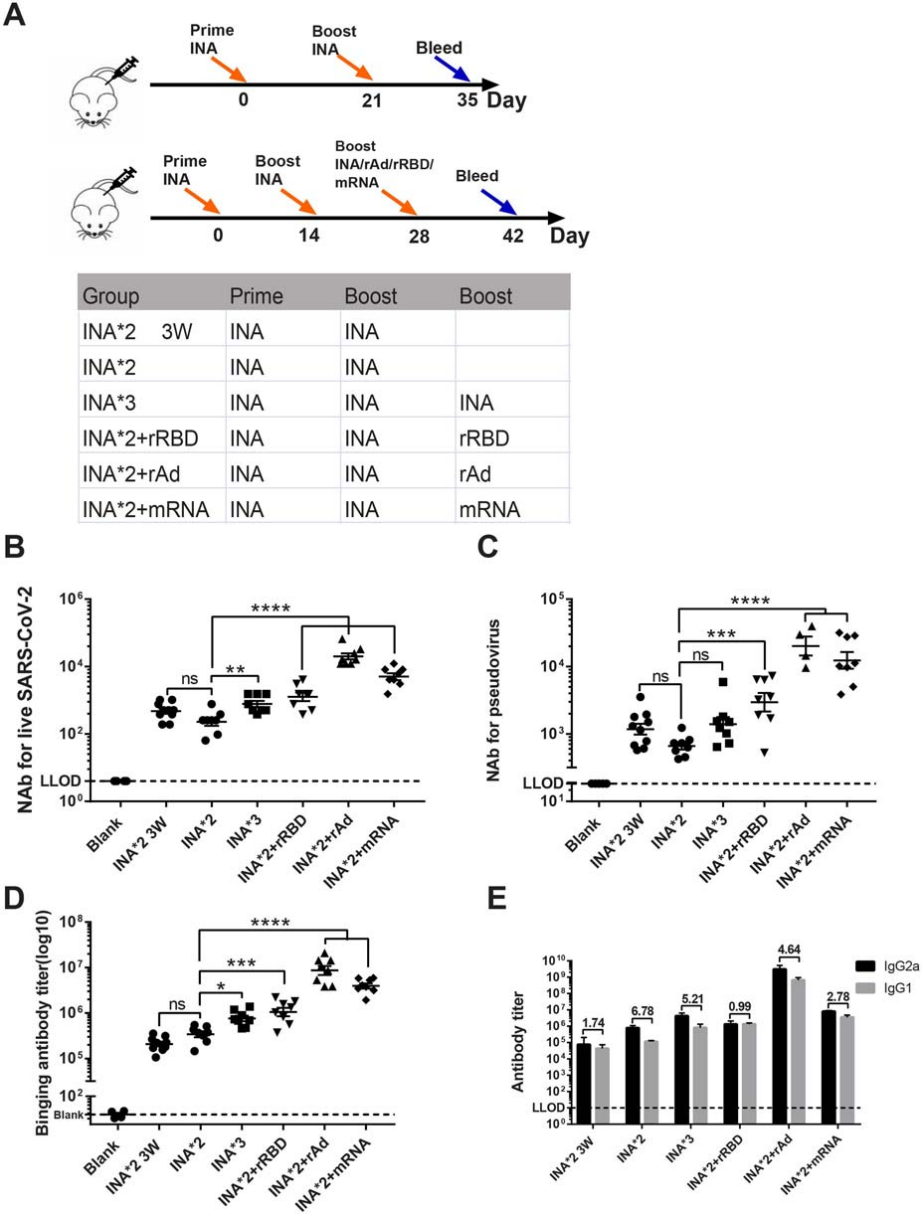
Humoral immune responses induced by homologous or heterologous prime boost regimens (A). Schematic representation of experimental protocol and immunization groups. Mice in six groups were immunized with different vaccines: INA*2 3w, INA*2, INA*3, INA*2 + rRBD, INA*2 + rAd, INA*2 + mRNA. (INA: inactivated vaccine, rAd: recombinant Ad5 vectored vaccine, rRBD: recombinant RBD vaccine, mRNA: mRNA-based vaccine). Blank control group were injected with PBS. (B, C). Sera NAb titres measured by live SARS-CoV-2 virus (B) and pseudovirus (C), the NAb titres were expressed as 50% inhibitory dilution (EC50) of serum. **(D)**. Spike-specific binding IgG titres were measured by ELISA. **(E)**. The titres of IgG subtypes were measured by ELISA, the ratios of IgG2a/IgG1 for each group were calculated and shown in chart. *N* = 8 per group, one spot represents one sample. One-way ANOVA was performed for B, C and D; two-tailed student’s t-test was performed for E. Bars represent the mean ± SD, ns: *p* > 0.05, *p < 0.05, **p < 0.01, ***p < 0.001, ****p < 0.0001.

To further improve protective immune responses of inactivated vaccines (BBIBPCorV), we boosted mice with a third dose of homologous (INA) or heterologous (rRBD, rAd, mRNA or rDNA) vaccines (Figure 3(A)). Mice were immunized intramuscularly at intervals of 14 days and sacrificed for NAb and binding antibody measurement 14 days after the last dose. The NAb GMT of three doses of INA regimen (INA*3) against liveSARS-CoV-2 was 784, elevated by 3-fold compared with two doses of INA regimen (INA*2) group. NAb against live virus were further increased in INA*2 + rRBD, INA*2 + rAd and INA*2 + mRNA group by 1.6-fold (GMT:1273; *p* = 0.81), 25.6-fold (GMT:20,066; *p* < 0.0001) and 6.4-fold (GMT:5017; *p* < 0.0001), respectively, compared with INA*3 group (Figure 3(B)). The NAb levels against live virus of INA*2 + rRBD, INA*2 + rAd and INA*2 + mRNA group were also compared with single dose of rRBD, rAd and mRNA showed in Figure 1. NAb GMT (live virus) of INA*2 + rRBD is nearly 2-fold higher than rRBD group, but without significant difference (*P* = 0.1370). The NAb GMT(live virus) of INA*2 + mRNA group is significantly higher than single dose mRNA group (*P*<0.0001), and same trend was found for INA*2 + rAd group and rAd group (*P* < 0.0001).

Similar results were obtained in pseudovirus NAb assay. For INA*3 regimen, the Nab GMT against pseudovirus was 1393, which was 2.1-fold higher than INA*2 regimen. Heterologous prime-boost regimens, INA*2 + rRBD, INA*2 + rAd and INA*2 + mRNA, induced NAb GMTs of 2949, 20,134 and 12,328, respectively, indicating that the pseudovirus NAb GMTs were further elevated between 2.12-fold to 14.6-fold when compared with the INA*3 group (Figure 3(C)). Binding IgG titres were also elevated in INA*2 + rRBD, INA*2 + rAd, and INA*2 + mRNA groups when compared to the INA*3 group (Figure 3(D)).

To elucidate the status of the balance of systemic Th1/Th2 type responses, we measured the IgG1 and IgG2a titres in mouse sera. The IgG2a titre was significantly higher in INA*2 + rAd group than INA*3 groups (*p* < 0.0001). There was no significant difference between INA*2 + rRBD, INA*2 + mRNA and INA*3 group. The IgG2a/IgG1 ratios were calculated (Figure 3(E)). Our results showed that the IgG2a/IgG1 ratios were higher in single INA vaccine groups, such as INA*2 (ratio: 6.78) and INA*3 (ratio: 5.21) groups, which is consistent with the IgG2a/IgG1 ratio data induced by one dose of vaccine as shown in Figure 1(E). For heterologous prime-boost regimens, INA*2 + rAd induced a IgG2a/IgG1 ratio of 4.64, higher than INA*2 + rRBD (0.99) and INA*2 + mRNA (2.78) group (Figure 3(E)).

To further investigate the role of time interval between the doses of vaccine in humoral immune responses, we immunized mice with 2 doses of INA at an interval of 21 days (INA*2 3w) (Figure 3(A)). Our results showed that the NAb titres of INA*3 3w group were slightly higher than INA*2 group. However, there was no significant difference between the two groups both in NAb and binding antibody titres (Figure 3(B–D)).

Taken together, our results indicate that boosting inactivated vaccine with a dose of heterologous vaccine could further improve humoral immune responses both for NAb and binding IgG when compared with homologous vaccine.

### Heterologous prime-boost with 2 doses of inactivated vaccine followed by a dose of either recombinant RBD, adenovirus vectored or mRNA vaccine effectively improves the Spike-specific T cell responses

**Figure 4.**
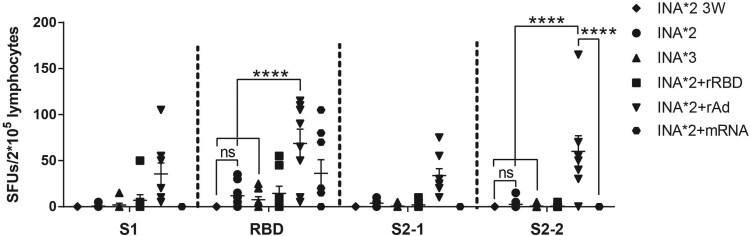
SARS-CoV-2 Spike-specific T cell responses induced by homologous and heterologous prime-boost regimens measured by INF-γ ELISPOT assay. Mice were sacrificed for measuring T cell responses. Isolated lymphocytes were stimulated with 4 spike peptide pools, and the IFN-γ secreting cells were quantified by ELISPOT assay. *N* = 6 per group, one spot represents one sample. One-way ANOVA was performed for comparison. Bars represent the mean ± SEM, ns: *p* > 0.05, ***p < 0.001, ****p < 0.0001.

To investigate the Spike-specific T cell responses induced by different regimens (see Figure 3(A)), splenic lymphocytes were collected and stimulated with four peptide pools spanning SARS-CoV-2 spike (Figure 2(A)). An ELISPOT assay was used for estimating the amount of IFN-γ secreted by the lymphocytes. Our results showed that the most recognized peptide pools originated from S1-RBD (aa: 325-576) (Figure 4), which was consistent with data shown in Figure 2. Our results further showed that there was no statistically significant difference between the SFUs/2 × 10^5^ cells induced by three doses of INA (INA*3) (S1-non RBD:1.88; S1-RBD:7.5; S2-1:0.63; S2-2:0.63) and those induced by two doses of INA (INA*2) (S1-non RBD:0.63; S1-RBD:11.88; S2-1:3.75; S2-2:2.5) (*P* > 0.05).

Interestingly, heterologous boost with rRBD, rAd or mRNA all elicited S1-RBD specific T cell responses successfully with SFUs/2 × 10^5^ cells of 14.38, 68.75 and 36.25, respectively. SFUs/2 × 10^5^ cells in INA*2+rAd group reached 35.63 for S1-non RBD, 68.75 for S1-RBD, 33.75 for S2-1, and 60 for S2-2, which were higher than the INA*3(S1-RBD: *p* < 0.0001) and INA*2(S1-RBD: *p* < 0.0001) group. The mean SFUs of INA*2 + rRBD, INA*2 + rAd and INA*2 + mRNA group were also compared with single dose of rRBD, rAd and mRNA (Figure 2). Our result showed that SFUs for RBD of INA*2+rAd group was slightly higher than single dose of rAd group (*p* = 0.7103), INA*2 + mRNA group were nearly 3-fold higher than single dose of mRNA group (*p* = 0.1901), and INA*2 + rRBD group was also higher than single dose of rRBD group (*p* = 0.1584), though none significant difference was found. Thus, a heterologous prime boost strategy consisting of 2-doses of inactivated vaccine followed by one dose of either recombinant RBD, adenovirus vectored or mRNA could effectively improve the Spike-specific T cell responses.

### Multiple cytokine analysis of homologous and heterologous boost post two-doses of inactivated vaccine

To further define the Th subtype of systemic T cell immune responses induced by the administration of heterologous vaccines following two doses of inactivated vaccines, a Meso Scale Discovery Assay (MSD) was conducted. Supernatants of splenic lymphocytes stimulated by four different spike peptide pools were collected for each regimen and pooled together. The samples were devoid of IFN-γ since it had bound to the ELISPOT plates. We analysed the samples for the presence of IL-2, IL-4 and IL-10 by MSD. IL-2 is mainly secreted by Th1 cells, while IL-4 and IL-10 are secreted by Th2 cells. IL-2 levels were highly improved after peptide stimulation in all vaccinated groups (from 0.55 to 14.82 pg/mL) when compared to blank (0.23 pg/mL). Remarkably, INA*2 + rAd induced higher levels of IL-2 (14.82 pg/mL) than other vaccinated groups, which was 4.16-fold of INA*2 group (3.56 pg/mL) (*p* = 0.0265) and 2.64-fold of INA*3 (5.61 pg/mL) (*p* = 0.1343) group. Moreover, the IL-2 levels were also elevated in group INA*2 + mRNA and INA*2 + rRBD compared with INA*2 (*p* = 0.6280, and *p* = 0.1148, respectively). IL-4 levels in mice administered with vaccines were not elevated post stimulation when compared with blank control (*p *> 0.05 for all regimens compared with blank). For IL-10, a modest increase was observed in vaccinated groups compared with blank control. However, there was no significant difference in the levels of IL-10 secretion between those seven vaccination regimens. The magnitude of secreted cytokine measured in supernatants collected was greater for IL-2 (mean for INA*3: 5.61; INA*2 + rRBD: 12.99; INA*2 + rAd: 14.82; INA*2 + mRNA: 9.70) than for IL-10 (mean for INA*3: 3.04; INA*2 + rRBD: 3.02; INA*2 + rAd: 4.60; INA*2 + mRNA: 4.20). Amongst these, INA*2 + rAd showed a higher potential to induce IL-2 responses than other groups. The descending order of the calculated IL-2/IL-10 ratios were: INA*2 + rRBD (4.30) > INA*2 + rAd (3.23) > INA*2 + mRNA (2.31) > INA*3 (1.85), indicating an obvious skew towards the secretion of Th1 cytokines in heterologous prime-boost vaccinated regimens.

## Discussion

Different vaccine platforms have been developed as countermeasures against COVID19. Although they target the same virus, SARS-CoV-2, the platforms differ vastly in immunogenicity. Reports on comparison of the immunogenic properties of COVID-19 vaccines studied under similar conditions using identical methods are sparse. In this study, we describe the immunogenic characteristics of vaccines developed by four different platforms. Humoral immune responses and T cell responses induced by inactivated vaccines (INA) (manufacturer 1), recombinant RBD vaccine (rRBD) (manufacturer 2), Ad5-vectored adenovirus vaccines (rAd) (manufacturer 3) and mRNA vaccine (mRNA) (manufacturer 4) in mice were evaluated. All the four vaccine candidates aforementioned elicited the production of NAb effectively with a seroconversion rate of 100%. NAb levels induced by one dose of rAd or rRBD were higher than one dose of INA and mRNA vaccine. Previously, Locci et al. investigated the immunogenicity of mRNA-LNP encoding full length S protein (Full S△furin mRNA) and recombinant SARS-CoV-2 RBD vaccine adjuvanted with AddaVax (rRBD-AddaVax) [31]. They reported an elevated NAb titre in mRNA group when compared with rRBD group, which was opposite to what we observed in our study. The differences in the results can be partly attributed to the dosage. While 30 μg mRNA vaccine and 10 μg recombinant RBD vaccine were used in their study, we tested 5 μg mRNA vaccine and 10 μg recombinant RBD vaccine. Moreover, the delivery system, antigen construction, and adjuvant used may also lead to the different results. mRNA vaccine used in our study was also based on LNP delivery system, while rRBD vaccine was a dimeric RBD vaccine adjuvanted by Alum. These results highlight the pitfalls of comparison of vaccines where the studies were not performed under identical conditions.

Heterologous prime-boost strategy has been pioneered one decade ago and proved to be effective in previous studies on vaccines against emerging virus such as HIV-1 [25], influenza [24] and SARS-CoV-2 [26,27]. Considering the distinct properties between inactivated vaccine and other kinds of vaccines, it’s rational to investigate the impact of homologous or heterologous vaccine boosting strategy based on the current clinical applied regimen of inactivated vaccine. In this study, we tested the 3-doses regimen for inactivated vaccine (manufacturer 1) in a mouse model in this study. Our result showed that the 3doses regimen improved the NAb level slightly than 2-doses regimen but not significantly. This is consistent with the reported phase 1–2 clinical trial data of inactivate vaccine developed by Wuhan institute of sinopharm, which demonstrated that 3-doses regimen (GMT:297) of did not significantly improve the NAb titres compared with 2-doses regimen (GMT:247) [13]. While, heterologous prime-boost with two-doses of inactivated vaccine followed by rRBD, rAd or mRNA all elicited significantly higher NAb levels than 2-doses regimen of single inactivated vaccine (Figure 3(B)). Interestingly, one-dose of mRNA we tested in this study in a mouse model did not showed any superiority in NAb levels against one-dose of inactivated vaccine as shown in Figure 1(B). Thus, The NAb responses were amplified when vaccinated with heterologous vaccines, and heterologous prime-boost could be an effective alternative approach to break the immunity bottleneck caused by homologous prime-boost.

Interestingly, though with relatively lower total IgG and NAb titre, inactivated vaccine induced a higher IgG2a/IgG1 ratio against S protein in mice (Figure 1(E)). For single dose vaccination, INA induced a IgG2a/IgG1 ratio of 23.92, significantly higher than other vaccines, the ratios of which stood between 0.53 and 1.74. For multiple doses, the IgG2a/IgG1 ratio induced by 2 or 3 doses of INA were also relatively higher than groups vaccinated by 2 doses of INA followed by a heterologous vaccine. The ratio of IgG2a/IgG1 reflects the balance of Th1-type/Th2-type immune responses in some degree. Higher IgG2a always mediated strong cell-mediated cytotoxicity (ADCC) effect and opsonophagocytosis by macrophages [32,33], which might provide an alternative approach eliminating virus. Notably, a heterologous boost with rAd after 2 doses of INA enhanced the induction of both the IgG2a and IgG1 by many folds. Specifically, IgG response induced by INA*2 + rAd was IgG2a biased with a IgG2a/IgG1 ratio of 4.64, which is higher than that of single rAd group with a ratio of 1.53 shown in Figure 1. Thus, a heterologous boost with adenovirus vectored vaccine after 2 doses of inactivated vaccine might confer greater protection against SARS-CoV-2 infections.

**Figure 5.**
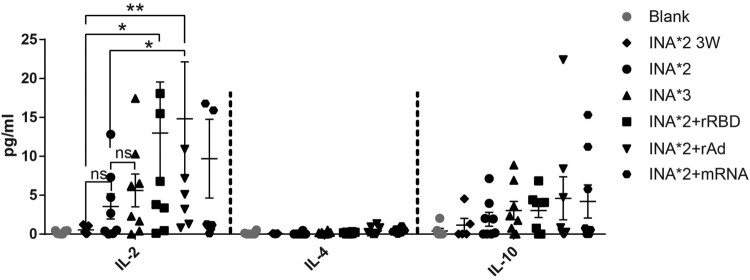
Multiplex cytokine analysis for homologous and heterologous prime-boost regimens. Lymphocytes isolated from mice kept on different immunization regimens were stimulated with 4 spike peptide pools. Supernatants were pooled by different groups or different peptide pools were collected. The IL-2, IL-4 and IL-10 in the supernatants were measured by MSD; the concentration of each cytokine (pg/ml) were represented by histogram. *N* = 6 per group. One-way ANOVA was performed for comparison. Bars represent the mean ± SEM, *p < 0.05, **p < 0.01, ns: *p* > 0.05.

T cell responses play an important role in combating SARS-CoV-2 infection [10,34]. In our study, the magnitude of Spike-specific IFN-γ secreting T cells induced by inactivated vaccine was relatively lower than other kinds of vaccines. The strong T cell responses induced by virus vectored and nucleic acid vaccines might benefit from the cytoplasm expression of antigen and the improving of MHC-I presentation process. “2-doses inactivated vaccine prime, adenovirus vectored or mRNA vaccine boost” regimens effectively increased the Th1-type T cell responses as indicated by higher levels of IFN-γ and IL-2 (Figures 4 and 5). We noticed that boosting with recombinant RBD or inactivated vaccine as a 3rd dose did not improve the magnitude of IFN-γ secreting T cells in comparison with 2 doses of inactivated vaccines. Amongst the two endogenous vaccines (rAd and mRNA) we tested in this study, rAd as a booster induced relatively higher IFN-γ secreting T cell responses. Actually, as we described in a previous report, heterologous prime with one dose of inactivated or recombinant RBD vaccine followed by adenovirus vectored vaccine elicited significantly higher T cell responses than two doses of inactivated or recombinant RBD vaccines [26]. Our results indicate that the combination of exogenous antigen with an endogenous antigen might effectively improve the targeted-antigen specific T cell responses, while the repeated use of exogenous antigens as boosters is not that effective. It should however be noted that the role of T cell responses in SARS-CoV2 infection has not been fully elucidated as yet. Safety issues arising out of activation of high numbers of CTLs, especially in people with known immune-related disorders and conditions should be given due consideration while scheduling heterologous vaccination strategies evoking strong T-cell responses.

In summary, we have characterized the humoral and cellular responses evoked by four COVID-19 vaccine platforms in mouse model. A heterologous prime-boost strategy consisting of 2 doses of inactivated vaccine followed by either a recombinant subunit, adenovirus vectored or mRNA vaccine increased NAb antibody titres and Th1-type T cell responses. Amongst, adenovirus-vectored vaccine and mRNA vaccine possess superior ability in improving NAb and T cell responses. Overall, our results demonstrated an applicable approach to improve immunogenicity of inactivated SARS-CoV-2 vaccines.

## Data and materials availability

All data generated in this study are available from the corresponding author upon reasonable request. Pseudoviruses used for NAb measurements can be obtained from the Chinese National Institution for Food and Drug Control. The live SARS-CoV-2 used for NAb measurements was isolated by Sinovac Research and Development Co., Ltd.
